# Reduction of nitrogen compounds in oceanic basement and its implications for HCN formation and abiotic organic synthesis

**DOI:** 10.1186/1467-4866-10-9

**Published:** 2009-10-22

**Authors:** Nils G Holm, Anna Neubeck

**Affiliations:** 1 Department of Geology and Geochemistry, Stockholm University, Stockholm, Sweden

## Abstract

Hydrogen cyanide is an excellent organic reagent and is central to most of the reaction pathways leading to abiotic formation of simple organic compounds containing nitrogen, such as amino acids, purines and pyrimidines. Reduced carbon and nitrogen precursor compounds for the synthesis of HCN may be formed under off-axis hydrothermal conditions in oceanic lithosphere in the presence of native Fe and Ni and are adsorbed on authigenic layer silicates and zeolites. The native metals as well as the molecular hydrogen reducing CO_2 _to CO/CH_4 _and NO_3_^-^/NO_2_^- ^to NH_3_/NH_4_^+ ^are a result of serpentinization of mafic rocks. Oceanic plates are conveyor belts of reduced carbon and nitrogen compounds from the off-axis hydrothermal environments to the subduction zones, where compaction, dehydration, desiccation and diagenetic reactions affect the organic precursors. CO/CH_4 _and NH_3_/NH_4_^+ ^in fluids distilled out of layer silicates and zeolites in the subducting plate at an early stage of subduction will react upon heating and form HCN, which is then available for further organic reactions to, for instance, carbohydrates, nucleosides or even nucleotides, under alkaline conditions in hydrated mantle rocks of the overriding plate. Convergent margins in the initial phase of subduction must, therefore, be considered the most potent sites for prebiotic reactions on Earth. This means that origin of life processes are, perhaps, only possible on planets where some kind of plate tectonics occur.

## Background

Geochemically plausible abiotic synthesis pathways and concentration mechanisms for nitrogen-containing molecules must eventually be found since nitrogen-based life is likely to have existed on Earth from early Archean onwards [1]. High ammonium contents (54-95 ppm) have been found in authigenic clays of the Isua supracrustal rocks of Western Greenland, suggesting that clays were major sinks of NH_4_^+ ^or other nitrogen compounds on the Earth's surface already at 3800 Ma [2]. Ward and Brownlee have argued that plate tectonics is necessary for the origin of life on terrestrial planets and have listed a number of reasons in support of their opinion [3]. However, one argument that they have never mentioned is the connection between plate tectonics, hydrothermal geochemistry and reduction of simple carbon and nitrogen compounds suitable for abiotic organic chemistry. In our opinion, the best location where such processes could occur would be at convergent margins during the early phases of subduction of oceanic plates.

### Palagonitization

Incipient alteration of mafic volcanic rocks (basalt; 45-52% SiO_2_) entails the palagonitization of glass with concomitant crystallization of authigenic layer silicates (e.g. smectites, double layer hydroxides) and zeolites [4-6]. Zeolites like phillipsite coexist with smectite and almost always with mafic glass [7]. The term palagonite is normally used in reference to a bulk sample of metabasite which contains a mixture of palagonitized glass, authigenic minerals like smectite, corrensite, zeolites, carbonates and Fe-Ti oxides and phosphates, as well as primary minerals like plagioclase feldspars, clinopyroxene and olivine [4]. Minerals with expanding-contracting sheet structures like double layer hydroxides (DLH) are capable of accommodating molecules of virtually any size and clamping the layer of sorbed reactant ions, and are found to have particularly high catalytic activity [1]. DLH may be formed by replacing a fraction of the divalent Mg^2+ ^in single layer magnesium hydroxide (brucite, Mg(OH_2_)) with common trivalent cations such as Al^3+^, Fe^3+ ^and Cr^3+^[1,8]. Brucite is a weathering product of olivine and pyroxene and constitutes the trioctahedral sheets of layer silicates (see section 'Serpentinization of olivine' below).

### Circulation of seawater in mafic rocks

**Figure 1:**
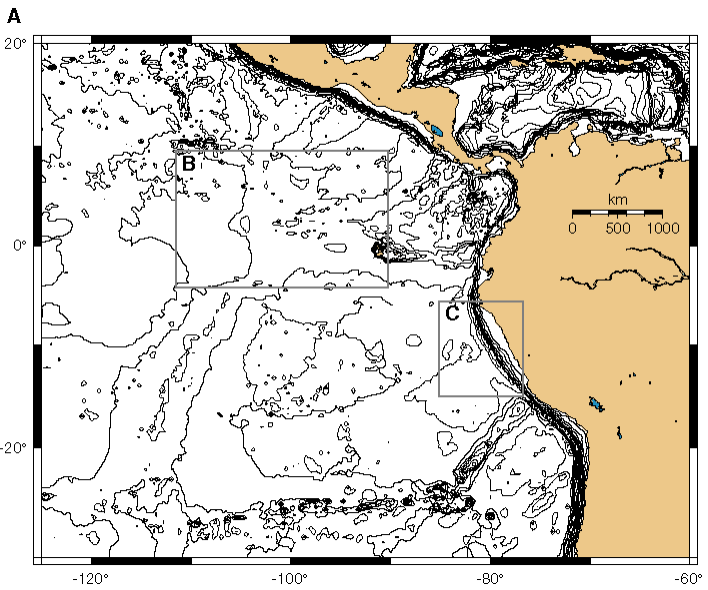
**Map showing the general areas of drill sites on both sides of the East Pacific Rise during Ocean Drilling Program (ODP) Leg 201 in the eastern equatorial Pacific Ocean (from D'Hondt et al., 2003 **[13]**, used with permission of the Integrated Ocean Drilling Program (IODP))**.

**Figure 2:**
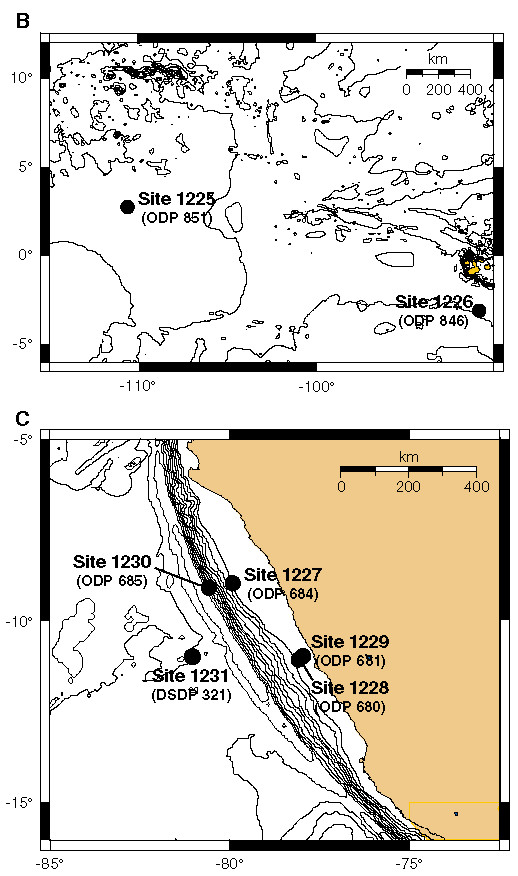
**Details of figure 1**. Previous DSDP/ODP site designations are in parantheses. The nitrate concentration of the sediment pore water of Site 1225 and Site 1231 are shown in figure 3. Site 1225 is located W of the East Pacific Rise spreading center and Site 1231 W of the subduction zone of the Peru margin (from D'Hondt et al., 2003 [13], used with permission of IODP).

**Figure 3:**
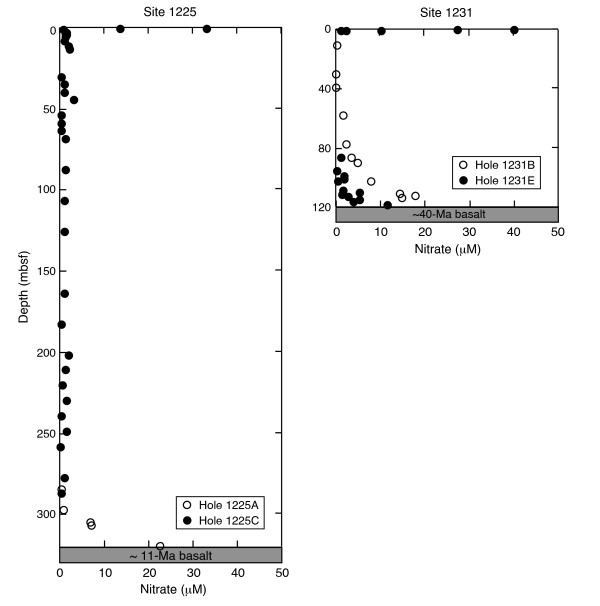
**Dissolved nitrate concentration in sediment pore fluids at open-ocean ODP Sites 1225 and 1231**. The nitrate values show that fresh seawater is channeled upwards into deep-sea sediments via the rocks underneath (from D'Hondt et al., 2003 [13], used with permission of IODP).

Seawater is constantly circulating through oceanic basement as a low-temperature fluid (< 150°C) [9]. Passive off-axis hydrothermal convection of seawater in older crust is in general a Rayleigh-Benard type circulation driven by the heat flow from the underlying, cooling crust [10]. Convection even in quite old crust is, however, in most cases still related to the original convection at the spreading ridge axis, although off-axis hydrothermal systems driven by exothermic hydration processes do exist in ultramafic rocks (< 45% SiO_2_). One example is the Lost City hydrothermal system near the Mid-Atlantic Ridge [11,12]. Results from the Ocean Drilling Program (ODP) Leg 201 (figures 1, 2 and 3) reveal that fresh seawater is channeled upwards into deep-sea sediments from the rocks underneath [13,14]. This happens still 40 Ma or more after formation of the basement and is illustrated by the concentration profiles of dissolved nitrate in sediment porewater from ODP Sites 1225 and 1231 (figure 3). Similar profiles have been obtained for dissolved sulfate. The circulation of modern seawater with oxidants like nitrate and sulfate into basement is driven by thermal advection with diffuse recharge and focused discharge through basement high to the seafloor [15]. This means that nitrogen that has been oxidized at the Earth's surface may be continuously transported by ocean water down into reducing environments of mafic or ultramafic rocks in oceanic basement. On the early Earth, oxidized nitrogen compounds (NO_2_^- ^and NO_3_^-^) may have been formed from N_2 _in a redox neutral atmosphere by lightning, corona discharge and impacts and subsequently transported by fluid circulation into reducing environments of the lithosphere [16].

### Nitrogen reduction in oceanic basement

**Figure 4:**
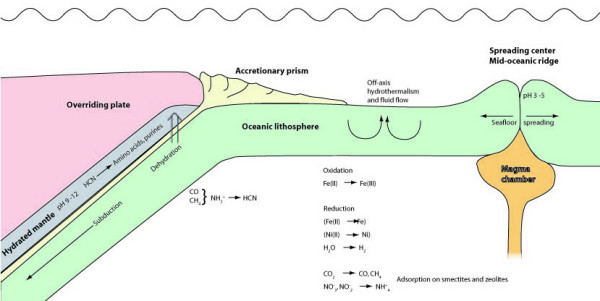
**Cartoon showing a cross section of oceanic lithosphere, extending from the spreading center to the subduction zone**. Off-axis hydrothermal flow in the oceanic lithosphere causes oxidation of Fe(II) to Fe(III) and reduction of water to molecular hydrogen. Some Fe(II) and Ni(II) is reduced to native metals. Carbon dioxide is reduced to carbon monoxide and methane, while nitrate and nitrite may be reduced to ammonium and adsorbed on smectites and zeolites. During early subduction the descending plate is heated and dehydrated. Adsorbed carbon monoxide and methane may react with ammonia and form hydrogen cyanide. The released fluid carrying hydrogen cyanide rises from an environment of relatively low pH into hydrated mantle rock of high pH. At the high pH hydrogen cyanide may form HCN oligomers as well as amino acids, purine bases, nucleosides and, perhaps, nucleotides.

Systems containing NH_3_/NH_4_^+ ^are more efficient in abiotic organic synthesis than those dominated by N_2 _in both aqueous and gaseous environments [16]. Among aqueous environments, hydrothermal systems represent regions of the highest NH_3 _conversion rates and stability on the Earth [17]. Hydrothermal experiments have shown that NO_2_^- ^and NO_3_^- ^are converted to NH_4_^+ ^more rapidly than N_2_[16]. Reduction of N_2_, NO_2_^- ^and NO_3_^- ^to NH_4_^+ ^is catalyzed by elemental Ni and Fe in the form of native metals or alloys. They can form in hydrothermal systems from Ni-containing rock-forming minerals like olivine and pyroxene [16]. Both native Fe and Ni as well as alloys of the two elements are very effective in converting NO_2_^- ^and NO_3_^- ^into NH_4_^+ ^at 200°C. However, at 70°C native Ni and Fe still effectively convert NO_2_^- ^and NO_3_^- ^into NH_4_^+^, whereas the reduction in the presence of FeNi alloys was insignificant [16]. During weathering of olivine and pyroxene in mafic rocks Fe(OH)_2 _may be formed as an intermediate phase in the partial oxidation of Fe(II) [18]. Fe(OH)_2 _is metastable with respect to magnetite and will convert to magnetite via a spontaneous reaction [19]. However, this conversion also creates a small amount of native iron that may assist in the reduction of NO_2_^- ^and NO_3_^- ^to NH_4_^+^. Dekov [20] has found tiny metallic particles consisting of Ni^0 ^in the hydrothermal sediments of the Trans-Atlantic Geotraverse (TAG) hydrothermal field, Mid-Atlantic Ridge. Oxidation-reduction processes involving simple C and N compounds in different parts oceanic crust and upper mantle have been summarized in figure 4.

### Ammonium adsorption on layer silicates and zeolites

Many layer silicates, like smectites, and zeolites have high cation exchange capacity (CEC). The CEC of minerals is generally determined in the laboratory by the uptake and release of ammonium ions (NH_4_^+^) of a 1 M ammonium acetate solution [21]. The CEC of zeolites like phillipsite is higher than that of smectite (450 mEq/100 g as compared to 60-150 mEq/100 g) [21,22]. NH_4_^+ ^may replace K^+ ^in silicates in hydrothermal environments and eventually form ammonium feldspar, buddingtonite [23].

### Zeolites as catalysts for the synthesis of organic nitrogen compounds

The adsorption properties of zeolites are very powerful, and particularly molecules with dipolar moments such as H_2_O, NH_3_, CO and HCN are particularly strongly adsorbed [24,25]. CO or formate, a hydrated form of CO, may be formed as an intermediate during the reduction of CO_2 _to CH_4 _in the presence of native Fe and Ni [26]. In experiments at temperatures of about 250-325°C, CO+ NH_3 _adsorbed on zeolites react to give HCN [24]. In the same experiments, several amino acids and the puric substance adenine have been found. Shapiro [27] has discussed problems in the prebiotic synthesis of adenine from HCN and popular ideas that adenine was easily formed and readily available on the early Earth. The background is that the purine coding elements of RNA, adenine in particular, can be easily synthesized by a one-step process from HCN [28,29]. One of Shapiro's [27] arguments against the availability of adenine on the early Earth is its hydrolysis at raised temperatures. For instance, according to experimental studies the half-life through nonenzymatic deaminination of adenine at 85°C would be 1.7 years [30]. However, most biochemical experiments trying to determine the thermal stability of organic compounds differ from traditional hydrothermal experiments in the sense that no attempts are made to control or measure important chemical or physical parameters such as oxidation state, pH, fugacities of dissolved gas species, or major and trace element compositions of the experimental systems [31]. As a comparison, recent geochemical studies have shown that adenine remains in detectable concentrations still after 200 hours at 300°C under fugacities of CO_2_, N_2 _and H_2 _representative of those in hydrothermal systems of the early Earth [32]. Another important feature of hydrothermal cells is that they are dynamic flow through systems. An organic compound that has been synthesized at high temperature will not necessarily remain within that environment for a long time.

One additional argument by Shapiro [27] against abiotic adenine formation on the young Earth was that such synthesis would require HCN concentrations of at least 0.01 M. Such concentrations in fluids would have been expected only under unique circumstances on the early Earth. However, since HCN is strongly adsorbed by zeolites it could be much more concentrated on surfaces than in solution. As an example, the adsorption properties of zeolites are so powerful that a 10^-4 ^atm partial pressure of CH_4 _suffices to fill up the zeolites cavities entirely with physically adsorbed molecules at liquid air temperature [24]. Since molecules with dipolar moments are still more strongly adsorbed, even lower activities of those would be necessary. In addition, HCN will outcompete H_2_O on the zeolite surfaces due to higher dipole moment [33].

### The importance of HCN

Hydrogen cyanide is central to most of the reaction pathways leading to abiotic formation of simple organic compounds containing nitrogen. HCN is likely to have been present in prebiotic hydrothermal environments because it is formed by a variety of processes driven by thermal energy [34]. HCN is, for instance, readily formed by reactions such as(1)(2)

These reactions are promoted by heat (geothermal), UV light, or electric discharges (lightning) [35]. Since the conversion of N_2 _to NH_3 _is relatively sluggish in off-axis type hydrothermal environments [16], pathway no. 2 is perhaps more likely to occur in mafic rocks than pathway no. 1. On the other hand, an additional possibility would be formation of HCN from CH_4 _and NH_3_, which is known to occur in the presence of aluminum oxide and silicate at high temperatures [36,37]:(3)

Shock [38] has calculated the concentrations of HCN from CO_2 _and N_2 _along paths in East-Pacific Rise type on-axis and off-axis hydrothermal systems. The initial fugacities of CO_2 _and N_2 _were set to 10 bars and 1 bar, respectively. In both cases the maximum concentration would be 10^-10^-10^-11 ^M HCN in an environment buffered by the fayalite-magnetite-quartz (FMQ) mineral buffer assemblage at temperatures of 250-400°C. Obviously, this would not be optimal conditions for HCN formation. However, recently LaRowe and Regnier [39] calculated the thermodynamic potential for the abiotic synthesis in hydrothermal systems of the RNA and DNA purine and pyrimidine bases as well as ribose and deoxyribose. The activities of precursor molecules (formaldehyde and hydrogen cyanide) required to evaluate the thermodynamics of biomolecule synthesis were computed using the concentrations of aqueous N_2_, CO, CO_2_, and H_2 _reported in the ultramafic Rainbow hydrothermal system on the Mid-Atlantic Ridge. Their results suggest that nucleobases at activities of 10^-2^-10^-6 ^M can be in equilibrium with a range of precursor molecule activities at 150°C and 500 bars, i.e. approximately under the conditions of hydrothermal flanks.

In order to participate in abiotic organic reactions HCN must first be concentrated. In addition to adsorption on mineral surfaces, one possibility is concentration to a reservoir of ferrocyanide at relatively low pH from which free HCN can be released upon local elevation of the pH [1,40]. HCN reacts with ferrous ions to give ferrocyanide, provided that the concentration of hydrogen sulphide is low [41]. The elevation of pH would lead to oxidation of Fe(II) and precipitation of the iron as FeOOH. This would avoid the 'Miller paradox', which refers to the side reaction of glycolonitrile (cyanohydrin of formaldehyde) formation from free HCN and ubiquitous formaldehyde [42]. Formaldehyde and HCN, if present in the same environment, tends to react to form stable glyconitrile. In natural environments, the occurrence of ferrocyanides in hydrothermal environments has been reported from the Kuril Islands and the Kamchatka Peninsula [43,44].

The self condensation of HCN in mildly basic solutions results in the formation of the tetramer diaminomaleonitrile (DAMN), which is a central intermediate in the formation of the purine ring and HCN oligomers [34]. Purines, especially adenine, are easy to form abiotically. Purines may be formed from HCN via two routes: one route is via HCN oligomers (that may also form amino acids); the second is via DAMN directly [45,46]. The abiotic formation of pyrimidines is much more problematic, very little pyrimidines have, for instance, been reported from carbonaceous chondrites [47]. It is known that double layer hydroxides promote the adsorption of cyanide and its self-addition to DAMN even down to low cyanide concentrations (0.01 M) [6]. The purine coding elements of nucleic acids are also much more strongly adsorbed to solids than the pyrimidines [48,49].

### Serpentinization of olivine

Olivine is one of the most easily weathered Fe(II) minerals and is particularly common in ultramafic rocks. Alteration of olivine in contact with water during hydrothermal circulation leads to 'serpentinization', a process in which Fe(II) in olivine in a side reaction is oxidized to Fe(III) coupled to reduction of water [50]. The entire process leads to the formation of serpentine, magnetite, molecular hydrogen and - during serpentinization at low temperature (less than about 315°C) - brucite [51,52]. Due to the formation of molecular hydrogen serpentinized ultramafic rocks are important environments for chemoautotrophic bacteria on the modern Earth. Brucite incorporates an increasing amount of Fe(II) with decreasing temperature, so the amount of Fe converted to magnetite (and H_2_O to H_2_) decreases with decreasing temperature below 315°C [52,53]:(4)

However, the stability of Fe-rich brucite depends on the activity of H_2 _in the system and, if the H_2 _activity decreases, Fe-bearing brucite will become unstable, decomposing to magnetite and brucite with a lower Fe content [52]:(5)

Therefore, in serpentinites initially formed at low temperatures, the destabilization of Fe-bearing brucite may result in a steady source of H_2 _in response to loss of H_2 _from the system [52]. A characteristic feature of many serpentinization systems is the high fluid pH, ranging up to values of about 12.6, because of the high solubility of the brucite [51]. Alkaline fluids are characteristic of deep aquifers of ultramafic rocks such as hydrothermal systems of ridge flanks (Lost City, Mid-Atlantic Ridge; pH 9-9.8 [11]) and non-accretionary supra-subduction zones (SSZ) (Mariana forearc; pH 12.6 [54]). By comparison, the measured values of pH for basaltic vent fluids at 25°C and 1 bar are in the range 3 to 4 for fluids venting from sediment starved ridge crests [38].

### Methane in layer silicates

Methane-rich plumes in deep ocean waters are known to be linked to hydrothermal circulation [55]. Fischer-Tropsch type (FTT) reactions are well known processes for converting CO_2 _to hydrocarbons (primarily CH_4_) by reaction with H_2 _and takes place on a large scale around mid-oceanic ridges [18,55]. Hydrocarbon generation through FTT is only possible if H_2 _is first generated. Production of molecular hydrogen in oceanic lithosphere is normally a result of serpentinization. It is well known that it is difficult to produce organic molecules directly from CO_2 _in abiotic synthesis experiments [18]. It is, however, easy to do it from CO and commercial Fischer-Tropsch reactions are normally optimized for the synthesis of hydrocarbons from CO and H_2_[34]. It is likely that native metals present in mafic rock like Fe and Ni and FeNi alloys reduce CO_2 _to CO or formate as an intermediate in abiotic organic synthesis [18].

It has been found that treatment with strong alkali releases methane, in particular, but also ethane and propane from both oxidized and reduced sediments [13,56]. The hydrocarbons could, in principle, have been formed either abiotically or biologically. The requirement to use strong base suggests that they are strongly sorbed to hydrophobic siloxane patches of the tetrahedral layer within the interlayer region of minerals like smectites [57,58]. Hydrocarbons that are formed abiotically on the flanks of mid-oceanic ridges will be adsorbed strongly on such secondary layer minerals. Once adsorbed, they will be carried passively along with the oceanic plate towards a subduction zone. Even though CH_4 _and NH_3 _(and probably HCN) will exist adsorbed on secondary minerals close to each other not much will happen with regard to organic chemistry until the initial phase of subduction of the plate starts. Thus, oceanic plates become conveyor belts of organic precursors from spreading centers to subduction zones.

### Organic processes during subduction of plates

Convergent margins in the form of subduction zones are the most dynamic regions on Earth. As the plate descends, fluids distilled from the plate influence and even control fundamental processes in the subduction zone (figure 4). The subducting plate, along with its fluids and altered igneous rock (and often sediments), interact with the overriding plate along the subduction zone. Fluids originating in the subducting plate will rise through the upper plate with its hydrated ultramafic mantle material (e.g. brucite) in the lower parts. The fluids may thus move from a relatively acidic environment in basalt altered to palagonite into an alkaline environment in serpentinized peridotite. The high pH promotes formation of, for instance, carbohydrates like ribose from simple organic compounds. It also supports the synthesis of amino acids and purine nitrogen bases as well as their condensation to nucleotides in the presence of phosphorylated ribose [59] (see 'Prebiotic implications of subduction' below). Hydrothermal fluid circulation in subducting basement also redistributes and extracts heat from the subduction zone, lowering temperatures far into the system and raising temperatures in the shallow subduction zone [60].

The Mariana forearc in the western Pacific Ocean is a non-accretionary forearc with numerous seamounts next to a deep ocean trench [61]. Secondary minerals at the South Chamorro Seamount include serpentine group minerals, brucite and magnetite [62]. The brucite of the South Chamorro Seamount serpentinites is never a pure Mg(OH)_2_, but always contains a significant amount of Fe(OH)_2_, thus indicating serpentinization well below 315°C [9,52,53]. The existence of Fe(OH)_2 _is of particular interest to prebiotic chemistry both because of the potential formation of native Fe and maintenance of low redox conditions (see section 'Nitrogen reduction in oceanic basement'), as well as increased stability of pyrophosphate in the presence of Fe(II) [63]. Chemical energy stored in pyrophosphates could have been used by primitive forms of life on the early Earth or for abiotic phosphorylation of carbohydrates and nucleosides [64].

The upper temperature limit for serpentinization at the Mariana forearc has been estimated to ~200-300°C [62]. Interstitial fluids of pH 12.6 associated with serpentinized mud at the South Chamorro seamount are enriched in dissolved carbonate, light hydrocarbons, ammonia and borate [54]. The interstitial fluids are, however, depleted in chlorinity as compared to seawater, which indicates a slab source of the fluids [61], probably due to smectite-to-illite transformation [58,65]. Pore fluids from Conical Seamount contain light hydrocarbons as well as organic acids, while fluid inclusions of the associated carbonate chimneys show the presence of light as well as longer chain hydrocarbons, aromatics and acetate [66]. These fluids derive from the subducting Pacific plate at an early stage of dehydration. After the fluids have been expelled from the subducting plate at moderate temperatures they are cooled down to a few °C on passage through the overriding Philippines plate/Mariana forearc.

Russell and coworkers have in a number of articles (see, for instance, Russell et al., 2005 [67]) proposed that modulated interactions between alkaline hydrothermal solutions and a weakly acidic early ocean created the conditions suitable for the reduction of CO_2 _to organic molecules and the onset of life. In the light of such ideas, a subduction zone setting like the Mariana forearc is optimal. Fluids would be distilled and squeezed out of secondary minerals into a relatively acidic environment (basalt/palagonite) (figure 4). CH_4 _and NH_4_^+ ^inherent in the fluids will react upon heating during subduction and form HCN. The fluids then rise through a strongly alkaline environment (serpentinized peridotite) into deep ocean water of, again, lower pH. Upon the increase of pH in hydrated mantle rocks, dissolved Fe(II) in ferrocyanide would be oxidized and precipitate as FeOOH. CN^- ^is, therefore, released and ready to participate in organic reactions. In his article, Shapiro [27] remarked that the rate of abiotic adenine formation from HCN is maximal at pH 9.2, which he, at the time, considered unlikely for environments of global distribution on the early Earth. In addition to adenine, amino acids may be synthesized in hydrothermal environments at fairly low temperatures (150°C) by Strecker type reactions (synthesis of amino acids from cyanide and aldehyde in the presence of ammonia) [68].

### Further prebiotic implications of the subduction

Pentoses like ribose can be formed by the formose reaction under alkaline conditions from simple organic precursors (formaldehyde and glycolaldehyde) [45,46]. Ribose is, like adenine, a major constituent of RNA. The formation of ribose proceeds by the stepwise condensation of formaldehyde to a dimer (glycolaldehyde), trimer, etc. Aldehydes can be formed directly from elemental carbon in the presence of water [69]. Elemental carbon in the form of graphite is common in peridotites [70]. The initial reaction of elemental carbon with water gives hydroxymethylene, which can rearrange to formaldehyde. A new hydroxymethylene molecule can then add onto the formaldehyde (and larger aldehyde molecules) and form glycolaldehyde. For a while, the formose reaction has been an outdated concept in prebiotic chemistry. A major reason for this is that the reaction proceeds at a constructive rate only under what was stated as naturally 'improbable' conditions, i.e. under highly alkaline conditions [9]. The recent discovery of alkaline hydrothermal systems in ultramafic settings, like the Mariana forearc, indicates that alkaline environments may be much more common on Earth than we thought just a few years ago. It has also been shown that borate minerals stabilize pentoses, particularly ribose [9,71,72]. The stability of the ribose-boron complex increases with increasing pH.

Even though a pH increase will destroy siloxane bonds between silicate minerals and methane, this is probably not the mechanism responsible for the release of methane and larger hydrocarbons in subduction zones. CH_4 _and NH_4_^+ ^bound to secondary minerals in the basaltic layer of the subducting plate will not experience the pH increase of the hydrated mantle of the overriding plate until they are already expelled from secondary minerals. Distillation, compaction, smectite-to-illite transition and similar diagenetic reactions are likely to be responsible for the liberation of the organic precursors [58,65]. However, even though conditions for abiotic organic synthesis may prevail in these environments, much of the organic precursors will pass relatively unaltered through the 'subduction factory' due to lack of activation energy, which is shown by the high concentrations of light hydrocarbons and ammonia in the Mariana forearc fluids [53]. Still, the possibility exists that the formose reaction is responsible for abiotic formation of ribose in natural settings and that this may occur in close vicinity to purine synthesis and, perhaps, phosphorylation processes [9]. The potential of pyro- and trimetaphosphate formation in hydrothermal environments of convergent margins has never really been evaluated. Once such condensed phosphates are available, phosphorylation of ribose or purine nucleosides is possible.

## Conclusion

• Nitrate and nitrite may be reduced to ammonium in oceanic basement in the presence on native Fe or Ni.

• The reduction is most efficient in hydrothermal environments.

• Ammonium may form hydrogen cyanide with carbon monoxide or methane.

• Hydrogen cyanide is an excellent starting compound for abiotic organic reactions.

• Oceanic plates are conveyor belts to the subduction zones of organic precursors formed in hydrothermal environments off-axis.

• The most potent prebiotic organic reactions on Earth occur in mafic rocks that are in the initial phase of subduction.

• The origin of life was, perhaps, only possible on planets with some kind of plate tectonics

## Competing interests

The authors declare that they have no competing interests.

## Authors' contributions

NH outlined the general structure of the paper and wrote the manuscript. AN provided the discussion on serpentinization of olivine and designed the cartoon in figure 4. Both authors have contributed to the discussion on prebiotic implications of subduction as well as read and approved the final manuscript.
